# Molecular mechanisms of resveratrol and its silver nanoparticle conjugate in addressing sepsis-induced lung injury

**DOI:** 10.1007/s00210-024-03058-y

**Published:** 2024-03-28

**Authors:** Hilal Üstündağ, Adem Kara, Songül Doğanay, Nezahat Kurt, Elif Erbaş, Ferdane Danişman Kalindemirtaş, İshak Afşin Kariper

**Affiliations:** 1 Department of Physiology, Faculty of Medicine, Erzincan Binali Yildirim University, Erzincan, 2400 Türkiye; 2 Department of Molecular Biology and Genetics, Faculty of Science, Erzurum Technical University, Erzurum, Türkiye; 3 Department of Physiology, Faculty of Medicine, Sakarya University, Sakarya, Türkiye; 4 Department of Biochemistry, Faculty of Medicine, Erzincan Binali Yildirim University, Erzincan, Türkiye; 5 Department of Veterinary Histology and Embryology, Faculty of Veterinary Medicine, Veterinary Medicine Basic Sciences, Erzurum, Türkiye; 6 Department of Science Education, Education Faculty, Erciyes University, Kayseri, Türkiye

**Keywords:** Polymicrobial sepsis, Resveratrol, Silver nanoparticles, Oxidative stress, P2X7 receptor, Inflammatory response

## Abstract

Sepsis is a life-threatening condition characterized by a systemic inflammatory response to infection. Despite extensive research on its pathophysiology, effective therapeutic approaches remain a challenge. This study investigated the potential of resveratrol (RV) and silver nanoparticle–enhanced resveratrol (AgNP-RV) as treatments for sepsis-induced lung injury using a rat model of polymicrobial sepsis induced by cecal ligation and puncture (CLP). The study focused on evaluating changes in oxidative status (TAS, TOS, and OSI) and the expression of inflammatory and apoptotic markers (IL-1β, TNF-α, P2X7R, TLR4, Caspase-3, and Bcl-2) in lung tissue. Both RV and AgNP-RV demonstrated potential in mitigating oxidative stress, inflammation, and apoptosis, with AgNP-RV exhibiting greater efficacy than RV alone (*p* < 0.05). These findings were corroborated by histopathological analyses, which revealed reduced tissue damage in the RV- and AgNP-RV-treated groups. Our study highlights the therapeutic potential of RV and, particularly, AgNP-RV in combating sepsis-induced oxidative stress, inflammation, and apoptosis. It also underscores the promise of nanoparticle technology in enhancing therapeutic outcomes. However, further investigations are warranted to fully understand the mechanisms of action, especially concerning the role of the P2X7 receptor in the observed effects. Nonetheless, our research suggests that RV and AgNP-RV hold promise as novel strategies for sepsis management.

## Introduction

Sepsis, a syndrome characterized by the body’s severe response to infection, has long been a significant burden on global health, leading to substantial morbidity and mortality. Its devastating effects stem from a dysregulated immune response, initiating a cascade of inflammation and often culminating in organ dysfunction (Singer et al. 2016). In particular, acute lung injury, a severe complication of sepsis, continues to be a key challenge due to its high prevalence and lack of effective treatments (Üstündağ et al. 2023a). The current therapeutic strategies, while valuable, are far from ideal, necessitating ongoing research into new approaches.

Resveratrol, a naturally occurring polyphenolic compound, has attracted considerable attention for its potential in combating various diseases. In the context of sepsis, the compound has shown promise due to its anti-inflammatory and anti-apoptotic properties. However, its therapeutic efficacy may be compromised due to issues like poor bioavailability and rapid metabolism (Rauf et al. 2018). Recently, efforts have been geared towards optimizing resveratrol’s delivery, with nanoparticle-mediated delivery emerging as a promising approach. Specifically, resveratrol-loaded silver nanoparticles could potentially enhance the effectiveness of resveratrol, increasing its stability and allowing controlled release (Sanna et al. 2014).

Understanding the molecular mechanisms behind sepsis-induced lung injury is vital for the development of potential therapies. The analysis of certain apoptotic and inflammatory markers such as Bcl-2, Caspase-3, P2X7R, TLR4, IL-1Β, and TNF-α has been proven to be crucial for this understanding (Youle and Strasser 2008; Riedl and Salvesen 2007; Di Virgilio et al. 2017; Kawai and Akira 2010; Dinarello 2009; Beutler 2004). These markers provide insight into the underlying cellular and molecular processes, potentially revealing therapeutic targets.

Acute lung injury (ALI), often accompanying sepsis, is marked by an overabundance of neutrophils in the lungs, elevated generation of reactive oxygen species (ROS), and a surge in pro-inflammatory cytokines, painting a picture of rampant inflammation and oxidative stress (Cinar et al. 2019). TAS, TOS, and OSI are key indicators of such oxidative stress, and their levels can provide valuable insight into the severity of sepsis-induced lung injury (Üstündağ et al. 2023a).

Markers like Bcl-2 and Caspase-3 offer a window into the apoptotic processes activated during sepsis. Bcl-2, a key anti-apoptotic protein, helps maintain cellular integrity, and its downregulation, as often seen in sepsis, can amplify apoptosis, contributing to organ dysfunction (Liu et al. 2022). Caspase-3, a significant executor of apoptosis, is characteristically upregulated during sepsis, promoting the apoptotic process (Shen et al. 2021).

In the inflammatory landscape of sepsis, markers like P2X7R, TLR4, IL-1Β, and TNF-α take center stage. The P2X7 receptor, activated by extracellular ATP, plays a role in immune cell function and apoptosis, with its expression often upregulated during inflammatory response (Kara and Ozkanlar 2023). TLR4, a key player in initiating the innate immune response, is implicated in the inflammatory surge seen in sepsis (Mollen et al. 2006). Pro-inflammatory cytokines IL-1Β and TNF-α, produced during the body’s immune response to sepsis, heighten the inflammatory response, potentially leading to severe organ damage, including the lungs (Üstündağ et al. 2023a; Cinar et al. 2019).

This study aims to assess the effects of resveratrol and resveratrol-loaded silver nanoparticles on sepsis-induced lung injury in male rats, with a particular focus on their impact on specific apoptotic and inflammatory markers. The novelty of this study lies in its approach of leveraging advanced nanoparticle-mediated delivery to potentially enhance the therapeutic efficacy of resveratrol, a development that could hold significant implications for the treatment of sepsis-induced lung injury. Our research seeks to illuminate potential therapeutic strategies against this condition by investigating the interplay of these markers and their response to the proposed interventions. By better understanding these molecular mechanisms, we inch closer to devising more effective therapies against sepsis-induced lung injury. This study, thus, bridges the critical gap between molecular understanding and therapeutic development, spotlighting the role of innovative delivery systems in the field of sepsis treatment.

## Materials and methods

### Animal and experimental groups

The experiment included twenty-eight Sprague–Dawley male rats aged between 8 and 10 weeks and weighing approximately 200–220 g. These rats were sourced and maintained at the Experimental Animal Laboratory of the Medicinal and Experimental Application and Research Center of Erzincan Binali Yıldırım University, Türkiye. The groups and treatments are presented in Table 1. The study was approved by the local animal care committee of Erzincan Binali Yıldırım University (2023/10).

**Table 1:** The experimental design

Groups	Applications (beginning of the study)	Intervention (2 h post-CLP)	Euthanasia time (end of the study)
Group I (SHAM; *n* = 7)	SHAM operation	0.5 ml saline	18 h post-operation
Group II (CLP; *n* = 7)	CLP procedure	0.5 ml saline	18 h post-operation
Group III (CLP + RV; *n* = 7)	CLP procedure	RV treatment, 30 mg/kg i.p. administered 2 h post-CLP	18 h post-operation
Group IV (CLP + AgNPs-RV; *n* = 7)	CLP procedure	AgNPs-RV treatment, 30 mg/kg i.p. administered 2 h post-CLP	18 h post-operation

### Induction of sepsis through CLP

The model of polymicrobial sepsis was established using the CLP method as described by previous studies (Wang et al. 1997). General anesthesia was applied to the rats by administering 100 mg/kg ketamine HCl and 15 mg/kg xylazine HCl intraperitoneally. The surgical field was then cleaned with a 10% povidone-iodine solution after shaving the abdomen. A longitudinal midline incision of about 2 cm was made in the abdominal area using dissection scissors to reach the peritoneum. The cecum was located and extracted through the incision. To ensure a consistent and reproducible sepsis model, the cecum content was pushed distally until filled with feces, then tied below the ileocecal valve using a 3.0 silk suture and punctured in two places with an 18G needle. After puncturing, the cecum, to contaminate the peritoneum with fecal content, was returned into the abdomen, which was subsequently closed with a 4.0 silk suture. Following this CLP procedure, a critical observation period of 18 h was established to monitor the progression of sepsis, culminating in the euthanization of the animals. This specific timeline was meticulously chosen based on literature evidence indicating the peak of inflammatory and oxidative stress responses within this timeframe post-sepsis induction euthanized (Wang et al. 1997; Üstündağ et al. 2023b), thereby ensuring that our observations captured the acute.

The four experimental groups were treated as follows:

- ***Group I (SHAM; n* = *7)***: These rats underwent a SHAM operation. Two hours post-operation, they were administered 0.5 ml saline. No further treatment was applied.
- ***Group II (CLP, n* = *7)***: These rats underwent a CLP procedure. Two hours post-operation, they were administered 0.5 ml saline. No further treatment was applied.
- ***Group III (CLP* + *RV; n* = *7)***: These rats underwent a CLP procedure and were administered 0.5 ml saline. Two hours post-CLP, they received RV treatment, at a dosage of 30 mg/kg i.p (Kolgazi et al. 2006).
- ***Group IV (CLP* + *AgNPs-RV; n* = *7)***: These rats underwent a CLP procedure and were administered 0.5 ml saline. Two hours post-CLP, they received AgNPs-RV treatment, at a dosage of 30 mg/kg i.p.

All treatments were administered at specific concentrations and intervals, which have been determined based on previous research studies. The study design, rat model, dosage, and administration schedule of treatments were carefully controlled to minimize variability and potential confounding factors.

### Drug preparation

The preparation of AgNPs and AgNPs-RV involved several steps. Firstly, solutions of 0.1 mM Ag(NO_3_), 4.3 × 10^ − 3 M trisodium citrate, and 2 × 10^ − 3 M sodium borohydride were prepared. A mixture was then created with 2 ml of the Ag(NO_3_) solution, 24 ml of trisodium citrate, and 24 ml of sodium borohydride. This mixture was heated at 60 °C for 15 min, followed by stirring at 90 °C for an additional 15 min. Stock solutions of resveratrol were prepared at a concentration of 20 mg/ml. For the AgNPs-RV solution, 2.5 ml of the AgNPs stock solution was combined with 10 ml of the resveratrol stock solution in a beaker. This mixture was then sonicated for 5 min at room temperature in an ultrasonic bath. All the materials used in the preparation of these solutions, namely silver nitrate, NaBH_4_, trisodium citrate, and resveratrol, were of analytical grade and purchased from Sigma Aldrich (St. Louis, MO, USA).

#### DLS and Zetasizer measurements

Nanosize analysis of AgNPs and AgNPs-RV samples was performed by dynamic light scattering (DLS) measurements using Zetasizer Nano ZS using 4 mW He – Ne laser operating at 633 nm wavelength and 173° detection angle. Zetasizer measurements were made at room temperature.

#### EDX-STEM analysis

For STEM and EDX analysis, nano AgNPs ve AgNPs-RV samples were dropped on glass substrate and coated with Au/Pd at 45 angstrom thickness by Polaron sc 7620 mini sputter coater device. Regarding liquid samples’ analysis, imaging was performed by dropping on glass substrates and putting on the device after drying.

#### FTIR analysis of AgNPs ve AgNPs-RV

FTIR analyses of the samples were investigated by a PerkinElmer Spectrum 400 spectrometer with 4 cm^−1^ resolution and 10 scans per spectrum.

### Biochemical analysis

For the purpose of our study, we utilized the right and left lungs differently. The right lung was employed for histopathological examinations, while the left lung was preserved for biochemical analysis. For this process, the left lung tissue was carefully harvested and immediately stored at − 80 °C until further use. Prior to the biochemical analysis, the lung tissue was homogenized in a buffer solution to obtain a consistent and representative sample. This homogenate was then subjected to a series of centrifugation steps to remove cellular debris and to yield a clear supernatant for analysis. The biochemical assessment included an evaluation of tissue oxidative stress markers.

### Tissue oxidative stress markers analysis

#### Measurement of total antioxidant status (TAS)

The total antioxidant status (TAS) was determined using the Erel method (Erel 2004). A commercial kit from Rel Assay Diagnostics (Gaziantep, Turkey) was used to measure the TAS levels in the lung tissue. This test is based on the reduction of dark blue-green colored ABTS (2,2-azino-bis 3-ethylbenzothiazolin6-sulfonic acid) radical to colorless reduced ABTS form by the antioxidants in the sample. The change in absorbance at 660 nm, which can be spectrophotometrically measured, is related to the sample’s total antioxidant status. The test was calibrated with a balanced antioxidant standard solution called Trolox Equivalent, a vitamin E analog. The test results were calculated using a 1 mmol TroloxEquiv./l standard. The %CV values for the test, reported by the manufacturer, were within ± 10%, with a range of 1.20–1.50 mmol/l. The results were expressed as mmol/l for serum.

#### Measurement of total oxidant status (TOS)

The total oxidant levels in both serum and tissue were determined using a commercial kit from Rel Assay Diagnostics (Gaziantep, Turkey). We used an automatic measurement method developed by Erel to measure serum TOS levels (Erel 2005). In this method, the oxidants present in the sample oxidize the iron ion-o-dianisidine complex into ferric ion. This oxidation reaction is amplified by the abundant glycerol molecules present in the reaction medium. In an acidic medium, the iron ion forms a colored complex with xylenol orange. The color intensity, which can be spectrophotometrically measured, is correlated to the total amount of oxidant molecules in the sample. The test’s %CV values were reported to be within ± 10%, with a range of 4–6 µmol/l by the manufacturer. The analysis was calibrated with hydrogen peroxide, and the results were expressed as µmol H_2_O_2_ Eq./l for serum.

#### Calculation of oxidative stress index (OSI)

The oxidative stress index was calculated by dividing the total oxidant status by the total antioxidant capacity (obtained using the Erel method). For calculating the OSI value, the unit of TAS was converted to µmol/l, and the formula TOS (µmol H_2_O_2_ Eq/l) / TAS (mmol/Trolox Eq/l)*100 was used.

### Histopathological assessment and evaluation procedure

Upon completion of the experiment, the lungs of the euthanized rats were preserved in a 10% neutral formaldehyde solution, where they were fixed for 72 h. The tissues were then processed, involving passage through graded alcohol and xylene series, before embedding in paraffin blocks. This was crucial in order to obtain uniform tissue sections for subsequent histopathological evaluations. Tissue sections of 5µ thickness were then meticulously cut using a Leica RM2125 RTS microtome.

Following this, the sections were stained with Crossman’s Modified Mallory’s Trichrome and Periodic Acid Schiff (PAS) methods. These staining procedures aimed to visualize various elements of tissue damage. The Crossman’s Modified Mallory’s Trichrome staining provided a clear differentiation between cells and surrounding connective tissue, while the PAS method allowed for the identification of polysaccharides such as glycogen, and mucosubstances like glycoproteins, glycolipids, and mucins in the tissues.

Histopathological evaluations were then performed based on key features of interest including pulmonary edema, vascular and alveolar characteristics, and bronchiolar pathology. These were scored on a scale of 0 (indicating normal conditions) to 3 (representing severe conditions), following the guidelines laid out by Passmore et al. (Passmore et al. 2018). Intravascular obstruction, inflammatory cell infiltration, pulmonary obstruction, alveolar septal thickening, the presence of amorphous matter, and the separation of bronchial epithelium were all taken into account during the evaluation (Table 2).

**Table 2:** Scoring system for lung histopathology

Score	Vascular characteristics	Extravascular and alveolar formations	Bronchiolar features
0	Normal	Normal	Absent
1	Mild erythrocyte obstruction in interstitium	Presence of mild inflammatory exudate	Mild infiltration of inflammatory cells
2	Vascular occlusion, moderate hemorrhagic areas	Moderate inflammation, moderate alveolar septal thickening	Moderate inflammation, sporadic separations in bronchioles
3	Diffuse hemorrhage, severe erythrocyte, and vascular obstruction	Severe inflammation, severe alveolar thickening	Loss of bronchiolar epithelium

A Zeiss AXIO Scope.A1 microscope, equipped with a computer and camera extension, was utilized for a detailed microscopic examination. This high-resolution imaging system allowed for accurate interpretations and documentation of the observed histopathological features.

### Western blot analysis

Before western blot analysis, the acquired lung tissue samples were kept at − 80 °C in a deep freezer. The lung tissue samples were weighted and crushed in nitrogen gas, treated with radioimmunoprecipitation (RIPA buffer, Ecotech Bio, Turkey), supplemented with protease and phosphatase inhibitors, and homogenized using a tissue lyser device (Qiagen, USA) at 30 Hz for 20 s to determine the relative protein expressions of Bcl-2, Caspase-3, P2X7R, TLR4, Il-1β, and TNF-α. A protein assay kit was used to quantify lung tissue’s total protein (Pierce BCA, Thermo Sci., USA). Thirty micrograms of protein was then put into the PVDF membrane after being separated by 10% SDS-PAGE. First, at room temperature, 5% bovine serum albumin was used to block the membranes for 90 min. Then, the membranes were incubated at 4 °C overnight with the appropriate primary antibodies (IL-1β (sc-52012, Santa Cruz), TNF-α (sc-52746, Santa Cruz), P2X7R antibody (11,144–1-AP, Proteintech), Caspase-3 (sc-56053, Santa Cruz), Bcl-2 (sc-7382, Santa Cruz), and TLR4 (AF7017, Affinity Biotech), and Beta-actin (sc-47778, Santa Cruz)). After primary antibody incubation, the PVDF membranes were washed with TBST and then incubated for an additional 90 min at room temperature with the second antibody (Santa Cruz, sc-2004/sc-2005) coupled to horseradish peroxidase. Then, the protein bands were captured using the enhanced chemiluminescence reagent Western ECL substrate (Thermo, 3405), visualized, and analyzed by Image Lab™ Software (Bio-Rad, Hercules, CA, USA).

### Statistical analysis

Data obtained from the study were presented as mean ± standard deviation (SD). Prior to any further statistical analysis, the normality of data distribution was confirmed. Subsequently, a one-way analysis of variance (ANOVA) was performed to evaluate the overall significance of differences between groups. In order to identify which specific group comparisons contributed to the observed overall ANOVA significance, a Duncan post hoc test for multiple comparisons was conducted. The threshold for statistical significance was set at *p* < 0.05. Hence, any results with a *p*-value below this threshold were deemed to demonstrate statistically significant differences.

## Results

### Characterization of the physicochemical properties of resveratrol-loaded silver nanoparticles

**Fig. 1:**
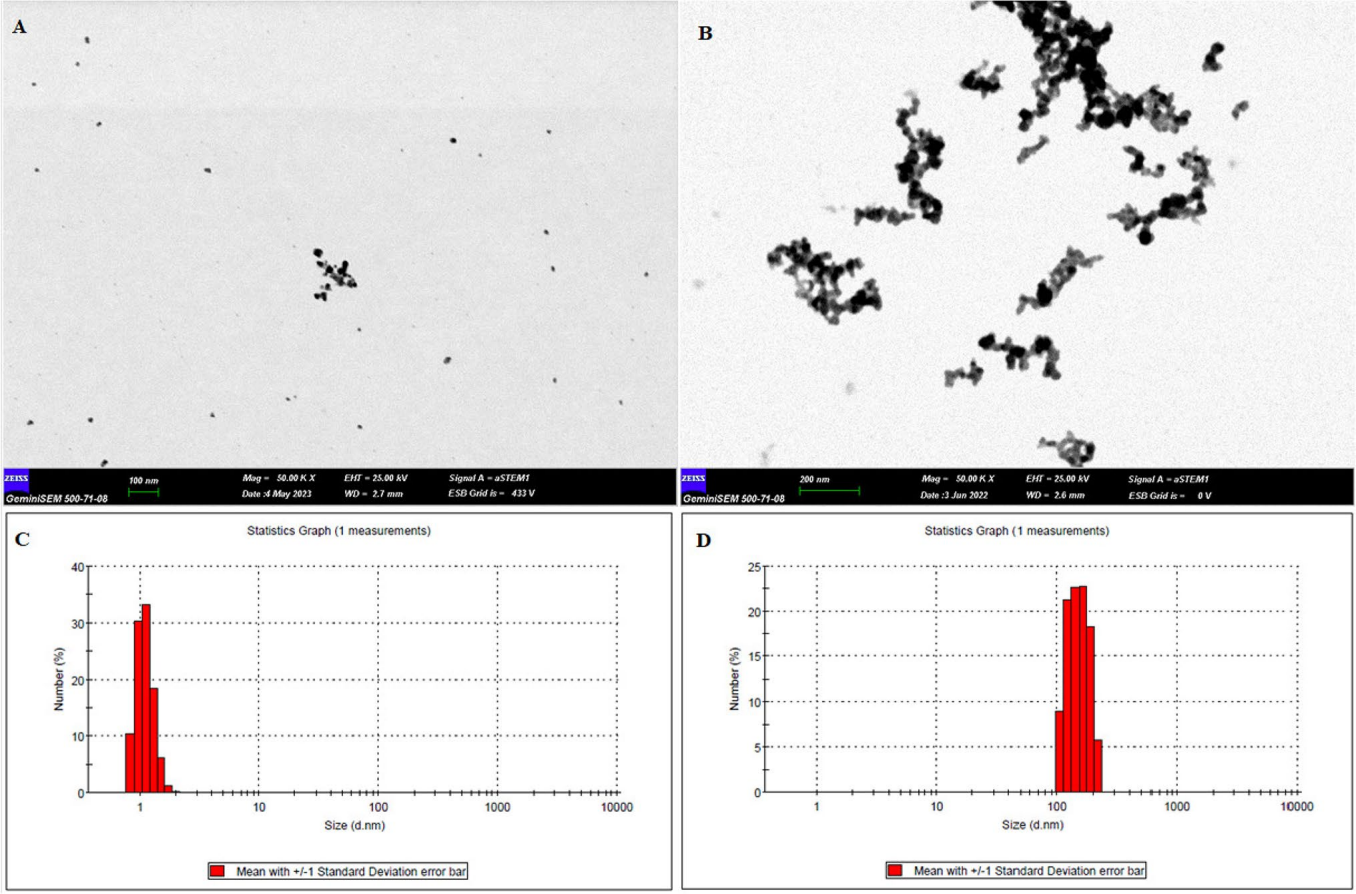
Characterization and analysis of AgNPs and AgNPs-RV. **A** Scanning transmission electron microscopy (STEM) imagery presenting the structure of AgNPs, with particle sizes ranging from 0.9 to 1.5 nm; **B** STEM images demonstrating the complex structure of AgNPs when amalgamated with RV, denoted as AgNPs-RV, exhibiting particle sizes of 95–160 nm; **C** Dynamic light scattering (DLS) assessment elucidating the particle size distribution of AgNPs, consistent with the nanometer-scale dimensions; **D** DLS analysis showcasing the varied particle size distribution detected when RV is incorporated with AgNPs, referred to as AgNPs + RV, indicating a substantial increase in particle size compared to pure AgNPs

The evaluation of the physicochemical characteristics of AgNPs and AgNPs-RV revealed distinct differences. The AgNPs exhibited a size range from 0.9 to 1.5 nm (as depicted in Fig. 1A), while the resveratrol-loaded AgNPs expanded to larger dimensions of 95 to 160 nm (illustrated in Fig. 1B). The zeta potential measurement showed a decrease from − 42.6 mV for AgNPs to − 45.4 mV for AgNPs-RV. Notably, the conductivity of the AgNPs decreased from 0.656 to 0.0625 mS/cm upon resveratrol loading. This phenomenon can be attributed to various solvent-related effects, such as the relaxation effect that limits conductivity in smaller AgNPs. This effect becomes more pronounced as the particle size increases to around 160 nm upon drug binding, as reflected in the changes in conductivity. The results from scanning transmission electron microscopy (STEM) (Fig. 1A and B) generally align with dynamic light scattering (DLS) measurements (Fig. 1C and D). However, some discrepancies exist, as AgNPs may appear larger during STEM evaluations due to static attraction between particles, a phenomenon that can occur as a result of solvent evaporation.

**Fig. 2:**
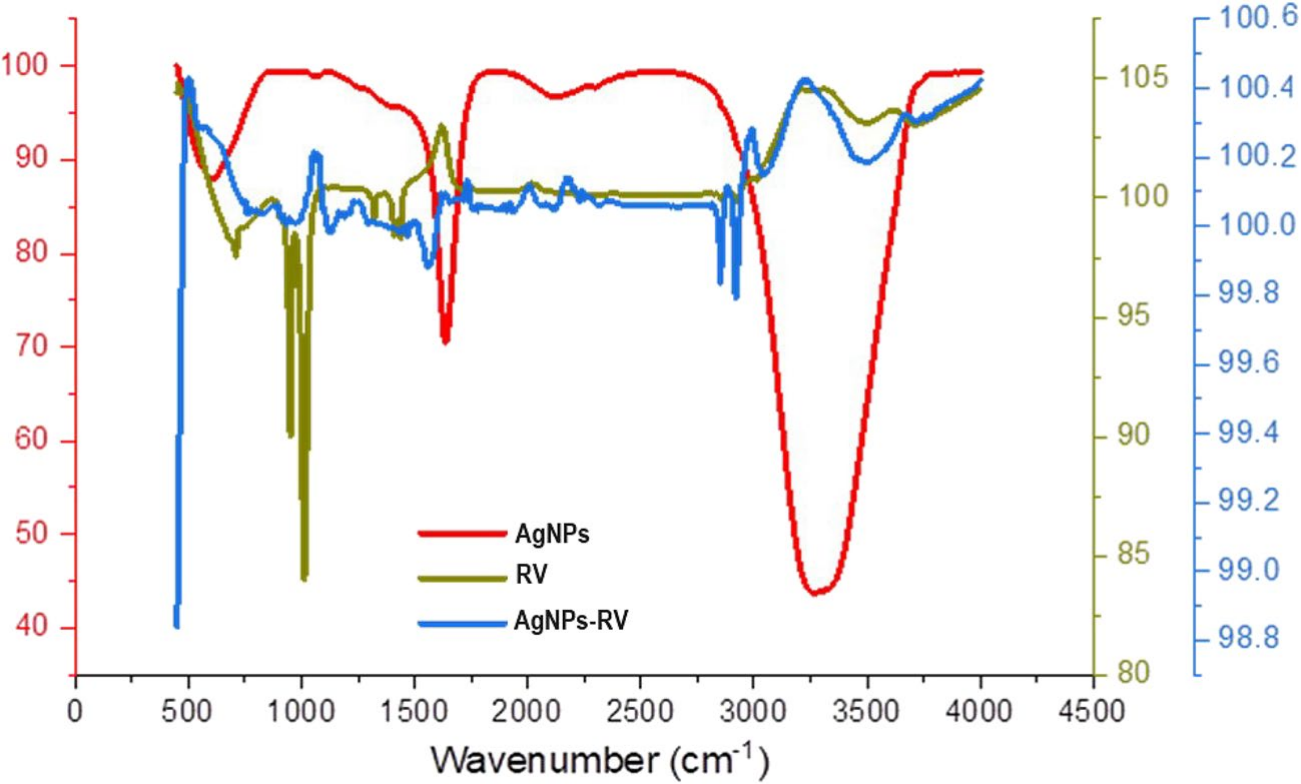
FTIR spectra of AgNPs and AgNPs-RV. This figure illustrates the FTIR spectra of AgNPs and AgNPs-RV, reflecting the vibrational peaks of various functional groups. The appearance of new peaks and shifts in the existing ones provides a clear indication of the successful integration of resveratrol onto the AgNPs, denoted by alterations in functional groups’ behavior. These spectral changes underscore the effective resveratrol loading, as well as the consequent physicochemical transformations in the resulting AgNPs-RV complex

Building on these findings, the Fourier transform ınfrared (FTIR) spectroscopic analysis further elucidated the physicochemical modifications brought about by the incorporation of resveratrol onto the AgNPs. Figure 2 portrays the FTIR spectra of both AgNPs and resveratrol-loaded AgNPs (AgNPs-RV). The figure highlights significant shifts and transformations in the vibration peaks of various functional groups after the introduction of resveratrol to the AgNPs.

Initially, the AgNPs display characteristic vibration peaks associated with specific functional groups such as − OH and − NH. Upon resveratrol integration, new peaks emerge, indicating the successful adhesion of resveratrol to the AgNPs. This is further substantiated by the appearance of new peaks that represent the characteristic functional groups of resveratrol.

### Tissue oxidative stress markers

**Fig. 3:**
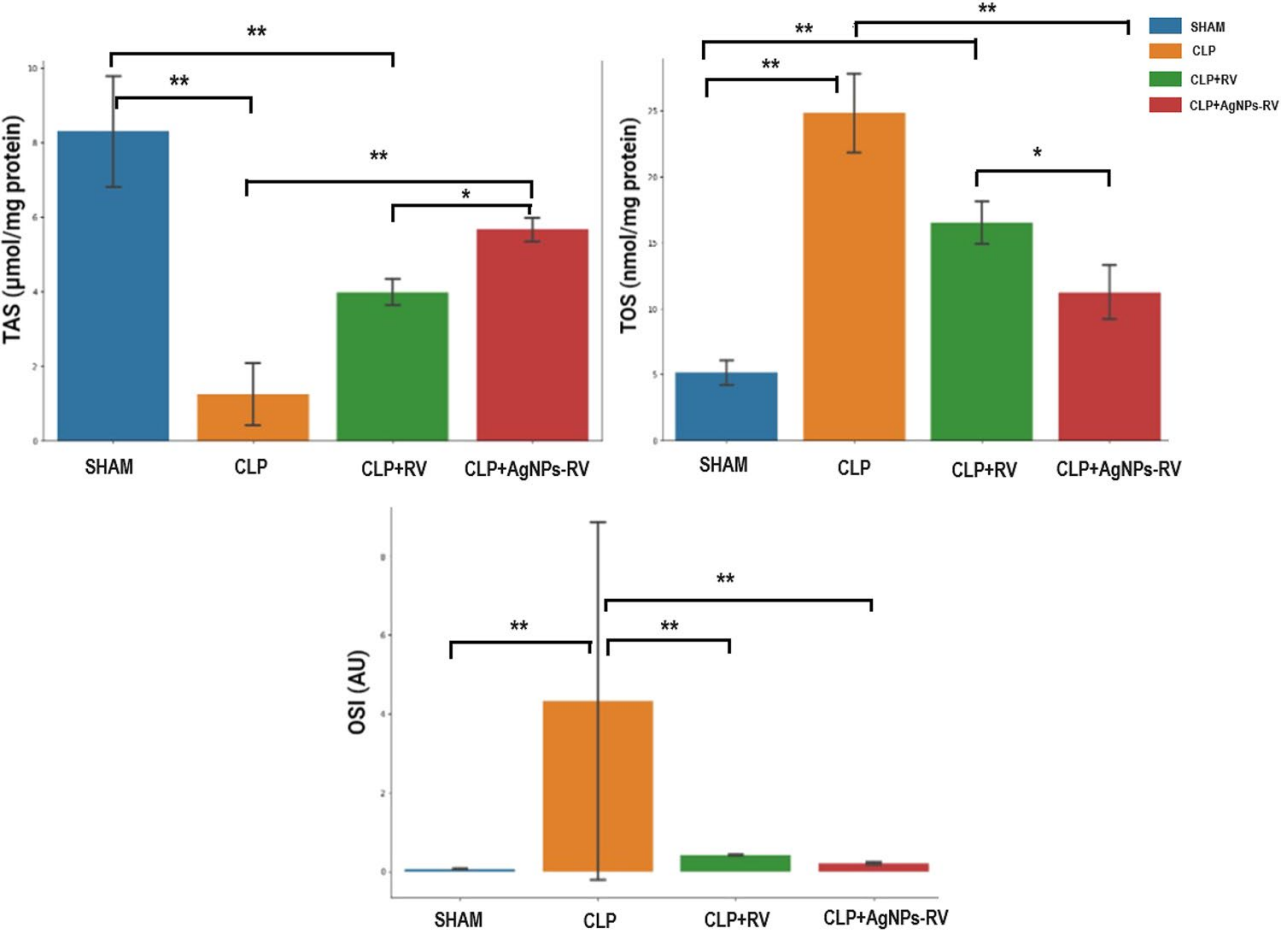
Comparison of TAS, TOS, and OSI levels across SHAM, CLP, CLP + RV, and CLP + AgNP-RV groups (*n* = 7 per group). TAS total antioxidant status, TOS total oxidant status, OSI oxidative stress index. Statistical significance: * for *p* < 0.05, ** for *p* < 0.01

Figure 3 provides a comparative overview of total antioxidant status (TAS), total oxidant status (TOS), and oxidative stress index (OSI) among the four groups under investigation.

In terms of TAS, the untreated CLP group exhibited significantly diminished levels when contrasted with the SHAM group (*p* < 0.001), indicating the introduction of severe oxidative stress due to the CLP procedure. However, upon treatment with RV and AgNP-RV, TAS levels in the respective groups recovered towards the range observed in the SHAM group, implying the efficacy of these treatments in mitigating the oxidative stress (*p* < 0.001).

The TOS levels followed an opposing trend, with the CLP group demonstrating a significant surge compared to the SHAM group (*p* < 0.001). This suggests an increased generation of oxidants following the CLP operation. Yet, both RV and AgNP-RV treatments significantly suppressed this surge (*p* < 0.001), highlighting their potential in reducing the oxidant production.

The OSI, a relative measure of oxidative stress, was dramatically escalated in the CLP group relative to the SHAM group (*p* < 0.001), further reinforcing the inference of intensified oxidative stress due to the CLP procedure. However, both RV and AgNP-RV treatment groups manifested significantly lower OSI values, nearing those of the SHAM group (*p* < 0.001), indicating their effectiveness in counterbalancing the oxidative stress introduced by the CLP operation. Notably, it appears that the AgNP-RV treatment group performed marginally better than the RV group in all the measured parameters.

### Histopathological findings

Upon histopathological examination of the lung tissues, the CLP group demonstrated significantly higher histopathological scoring compared to the SHAM group (*p* < 0.05). This highlights the severe histopathological alterations brought on by the CLP procedure. However, the treatment groups—CLP + RV and CLP + AgNP-RV—exhibited a substantial reduction in these scores compared to the CLP group (*p* < 0.05). This indicates that both treatments were successful in ameliorating the histopathological damage associated with the CLP procedure.

**Fig. 4:**
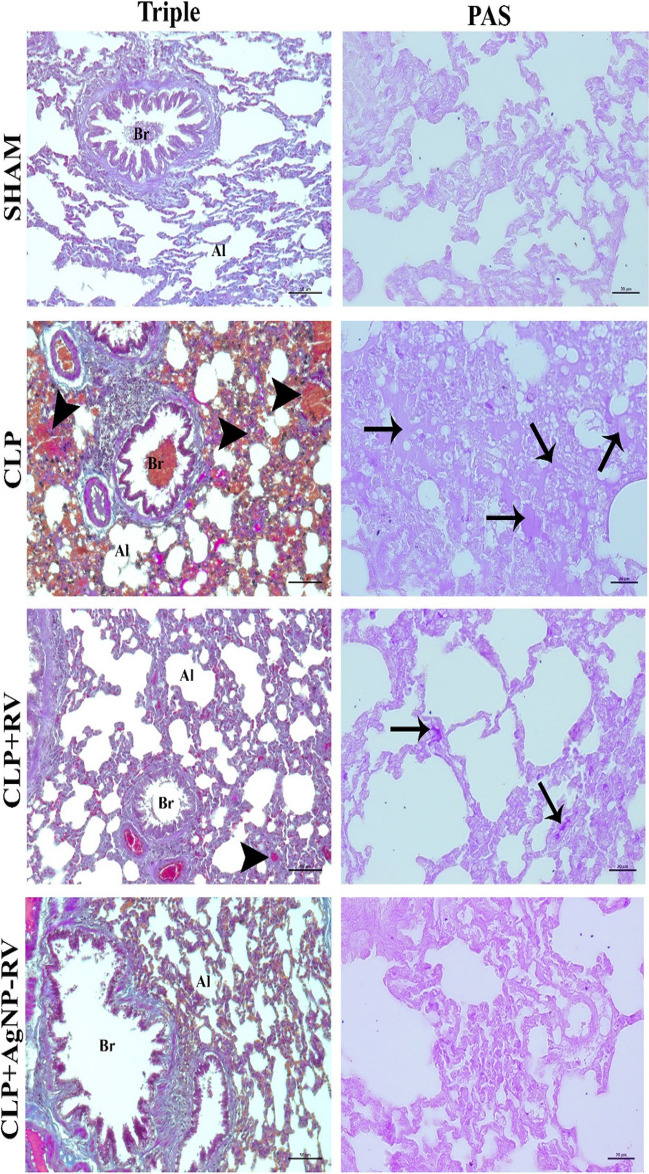
Micrographs of lung tissues from all groups (*n* = 7 per group) (Trichrome staining magnification × 200, PAS staining magnification × 400). Br bronchiole, Al alveolus; arrowhead, alveolar hemorrhage, arrow, collagen accumulation

Histopathologies of the lung tissues from all groups are presented in Fig. 4, with the corresponding histopathological scores detailed in Table 3.

**Table 3:** Histopathological scores of lung tissues from all groups. The scores were evaluated in terms of vascular characteristics, extravascular and alveolar formations, and bronchiolar features

Group	Vascular characteristics	Extravascular and alveolar formations	Bronchiolar features	Average score
SHAM	0.60 ± 0.54^a^	0.83 ± 0.40^a^	0.66 ± 0.51^a^	0.66 ± 0.16
CLP	2.33 ± 0.81^b^	2.16 ± 0.98^b^	2.00 ± 0.89^b^	2.16 ± 0.16
CLP + RV	1.66 ± 0.51^c^	1.50 ± 0.54^c^	1.16 ± 0.75^c^	1.44 ± 0.25
CLP + AgNP-RV	1.50 ± 0.54^c^	1.66 ± 0.40^c^	1.00 ± 0.63^c^	1.22 ± 0.25

Letters (^a, b, c^) next to histopathological scores in each column differentiate statistical significances among groups at *p* < 0.05. Scores marked with the same letter do not differ significantly, indicating analogous histopathological impacts within these groups. In contrast, scores with distinct letters have significant differences, underscoring the diverse effects of sepsis and treatment interventions on lung histopathology

The scores for vascular characteristics, extravascular and alveolar formations, and bronchiolar features were all significantly elevated in the CLP group compared to the SHAM group (*p* < 0.05). This underscores the detrimental effects of the CLP procedure on the structural integrity of lung tissues. Conversely, both RV and AgNP-RV treatments demonstrated significant reductions in these scores, indicating their potential in reversing the histopathological damage.

Remarkably, the AgNP-RV group scored slightly lower than the RV group, suggesting slightly better histopathological outcomes when utilizing resveratrol-loaded silver nanoparticles.

### Relative expressions of Bcl-2, Caspase-3, P2X7R, TLR4, IL-1β, and TNF-α

Our study revealed significant alterations in the expression of crucial apoptotic and inflammatory markers in the lung tissues of male Sprague–Dawley rats subjected to CLP.

The expression of Bcl-2, an anti-apoptotic protein, was prominently expressed in the control (SHAM) group. Following the induction of sepsis (CLP group), Bcl-2 expression significantly plummeted, indicating a considerable increase in cell apoptosis. Remarkably, treatment with resveratrol (CLP + RV group) and resveratrol-loaded silver nanoparticles (CLP + AgNPs-RV group) led to a marked resurgence in Bcl-2 levels, suggesting a potential protective effect against sepsis-induced apoptosis.

Conversely, the expression levels of pro-apoptotic and pro-inflammatory markers Caspase-3, P2X7R, TLR4, IL-1β, and TNF-α were found to be significantly escalated in the CLP group, which was indicative of the potent inflammatory response triggered by sepsis. Nevertheless, these levels were considerably mitigated upon treatment with resveratrol and resveratrol-loaded silver nanoparticles, implying their potential anti-inflammatory effects in the context of sepsis. In the SHAM group, these markers were found to be at their lowest levels, consistent with the absence of sepsis-induced inflammation.

**Fig. 5:**
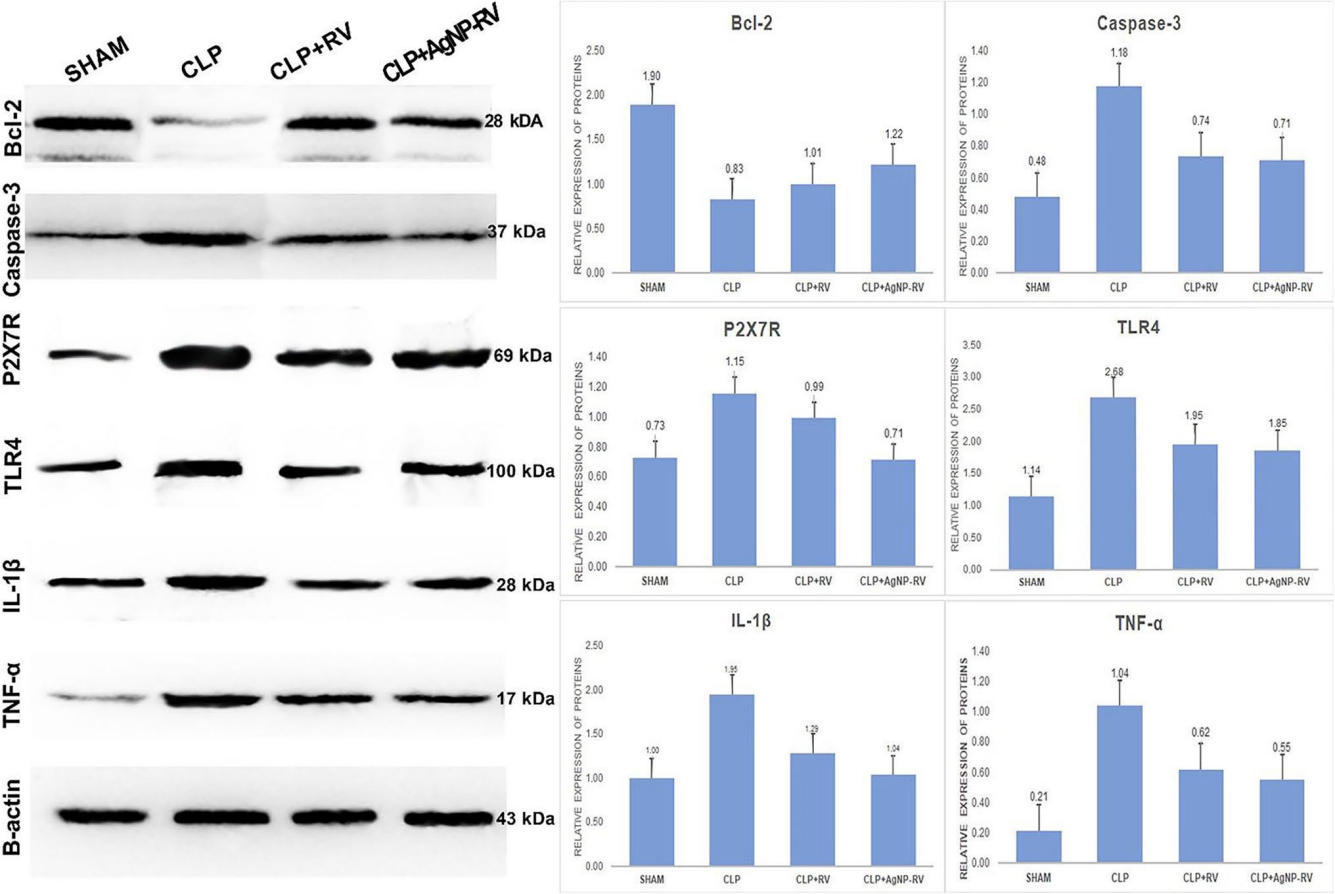
Relative protein expression levels of SHAM, CLP, CLP + RV, and CLP + AgNP-RV groups (*n* = 7 per group) lung tissues for Bcl-2, Caspase-3, P2X7R, TLR4, IL-1β, and TNF-α proteins

Figure 5 illustrates the relative protein expression levels of the markers analyzed for each group, providing a comparative visual representation of the protective effects conferred by resveratrol and resveratrol-loaded silver nanoparticles on sepsis-induced lung damage.

## Discussion

In the present study, we investigated the therapeutic potential of RV and AgNP-RV in mitigating inflammatory lung injury in a CLP-induced polymicrobial sepsis rat model. The focus of our examination was on the influence of RV and AgNP-RV on the sepsis-induced oxidative and inflammatory response in lung tissue. The oxidative status was assessed through the determination of TAS, TOS, and OSI levels. We further evaluated the impact of RV and AgNP-RV on the relative expressions of IL-1β, TNF-α, P2X7R, Caspase-3, and Bcl-2 key mediators in the inflammatory response and apoptosis pathway.

To substantiate these molecular analyses, we also conducted histopathological evaluations to understand the morphological changes in the lung tissue. The collective findings from these multifaceted analyses provide a comprehensive understanding of the therapeutic efficacy and the underlying mechanisms of RV and AgNP-RV in the context of sepsis-induced lung injury.

The pathophysiological processes underlying sepsis are intricately complex and involve a cascade of oxidative and inflammatory responses. The early stages of sepsis are characterized by a state of hyperinflammation, driven by the systemic production of inflammatory cytokines, such as IL-1, IL-6, and TNF-α (Ayala et al. 2003). This so-called cytokine storm can precipitate severe organ damage, and in certain cases, result in mortality. TNF-α, in particular, a multifunctional cytokine initially produced by lipopolysaccharide-induced monocytes and macrophages, plays a pivotal role in the inflammatory cascade and contributes significantly to the exacerbation of lung injury (Giebelen et al. 2007). In this context, our previous research has demonstrated the effectiveness of RV and AgNPs + RV treatments in modulating procalcitonin (PCT) and presepsin (PRSN) levels, which are significant markers of bacterial infection and systemic inflammation in sepsis (Üstündağ et al. 2023b). These findings highlight the potential of these treatments to not only counteract the cytokine surge but also to mitigate the associated organ damage by targeting these essential biomarkers.

In this context, our study observed increased levels of TAS, TOS, and OSI in the CLP-induced sepsis model. These measures respectively denote the overall antioxidant capacity, the oxidant status, and the oxidative stress balance within an organism, and their alteration in sepsis signifies a profound shift in the body’s redox homeostasis. The escalated TOS and OSI values highlight the excessive production of ROS and free radicals, which are known defense mechanisms against bacterial infections (Victor et al. 2004) and are particularly accentuated during sepsis (Di Meo et al. 2016). Indeed, previous studies corroborate our findings, demonstrating increased lipid peroxidation in sepsis patients (Takeda et al. 1984), along with a concomitant decrease in antioxidant enzymes (Goode et al. 1995). This surge in oxidant levels culminates in cellular damage by affecting proteins, lipids, and nucleic acids, thereby leading to endothelial dysfunction (Üstündağ et al. 2023c). Therefore, our findings not only align with existing literature but also underscore the value of antioxidant treatment protocols as potential therapeutic interventions against sepsis-induced lung damage. In view of this, the observed amelioration of TAS, TOS, and OSI levels by RV and AgNP-RV administration in our study suggests their potential role as therapeutic agents. By reducing oxidative stress and reestablishing redox balance, these compounds might mitigate tissue damage and potentially improve outcomes in sepsis-induced lung injury.

In our study, we specifically evaluated Bcl-2, a paramount anti-apoptotic protein, due to its critical role in controlling the balance between cell survival and death. In the control group (SHAM), Bcl-2 exhibited prominent expression, which can be attributed to the healthy, non-septic state of the subjects. However, sepsis induction (CLP group) precipitated a significant plunge in Bcl-2 expression, providing clear evidence of escalated cell apoptosis in the septic state. This is congruent with prior research underlining an inverse correlation between Bcl-2 expression and apoptotic activities, especially pertinent in septic conditions (Hotchkiss et al. 1999; Imai et al. 2008; Leonard et al. 2003). Intriguingly, our experimental interventions, resveratrol treatment (CLP + RV group), and resveratrol-loaded silver nanoparticle treatment (CLP + AgNPs-RV group) seemed to subvert this sepsis-induced apoptotic trajectory. These treatments prompted a remarkable recovery in Bcl-2 levels, suggestive of their potential to confer protection against sepsis-induced apoptosis. The observed anti-apoptotic influence could be rooted in the well-documented anti-apoptotic properties of resveratrol (Leonard et al. 2003; Kim et al. 2013). Thus, our findings enhance the understanding of the anti-apoptotic mechanisms mediated by resveratrol and its nanoparticle-enhanced version, setting a promising precedent for future therapeutic strategies against sepsis-induced lung injury.

Contrary to the expression patterns observed for anti-apoptotic markers, we found that pro-apoptotic and pro-inflammatory markers Caspase-3, P2X7R, TLR4, IL-1Β, and TNF-α saw a considerable surge in the CLP group, a reflection of the pronounced inflammatory response instigated by sepsis. The rise in these markers is indicative of an activated apoptotic pathway, driven by the death-inducing stimulus of sepsis (Hotchkiss et al. 2009; Fink and Warren 2014). Interestingly, our findings illuminate the therapeutic potential of resveratrol, a polyphenolic compound known for its anti-inflammatory and anti-apoptotic properties (Cottart et al. 2010; Truong et al. 2018), in alleviating sepsis-induced lung damage. We observed that resveratrol, both in its native form and encapsulated within silver nanoparticles, could substantially mitigate the sepsis-induced changes in the expression of Bcl-2, Caspase-3, TLR4, IL-1Β, and TNF-α. This revelation offers a window into the role of resveratrol in modulating both apoptotic and inflammatory responses in the context of sepsis. What is particularly remarkable, while silver nanoparticles are known for their antibacterial qualities (Rai et al. 2009) can improve drug bioavailability (Conde et al. 2012), the observed improvements in oxidative and inflammatory markers in the lung tissue for AgNPs-RV suggest an enhanced delivery and action of resveratrol when carried by AgNPs.

The multifaceted pathophysiology of sepsis unfolds through the intricate coordination of numerous molecular and cellular events in the inflammatory response. Amid this cascade, the P2X7R emerges as a key player, notable for its significant role within a complex pathway implicated in inflammation and immune responses (Kara and Ozkanlar 2023; Di Virgilio 2007). P2X7Rs are co-activated with LPS-sensitive TLR4, NLRP3, and pro-IL-1β-degrading caspase-1, facilitating IL-1β release (Ren et al. 2021). This pathway indicates the interplay between P2X7R and essential components of inflammatory and immune responses. In fact, P2X7R signaling, which is mediated by intracellular and extracellular Na^+^, K^+^, and Ca^2+^ fluxes (Panicucci et al. 2020), plays a vital role in averting alveolar epithelial cell death and hence modulates the severity of lung inflammation (Guo et al. 2014). The increasing expression of P2X7R in lung tissue during systemic LPS induction underlines its importance during sepsis-related lung inflammation. As such, strategies focusing on the inhibition of P2X7R have been devised as novel therapeutic interventions.

In the present study, we shed light on the capability of RV and AgNP-RV to inhibit P2X7R expression and activation in a polymicrobial sepsis model. The intensification of P2X7R expression and activation subsequent to sepsis triggers the production of pro-inflammatory cytokines (IL-1β and TNF-α), thereby inducing tissue damage (Alves et al. 2013; Mehta et al. 2014; Bartlett et al. 2014). Our findings reveal that both RV and AgNP-RV are able to suppress this cytokine production, thereby mitigating the inflammatory response, and indicating a potentially efficacious role in managing sepsis.

We observed that both RV and AgNP-RV could suppress this cytokine production and mute the inflammatory response, indicating a potentially efficacious role in sepsis management. Moreover, our research discerned a superior efficacy of AgNP-RV compared to RV alone. This supports the hypothesis that nanoparticles can enhance the effectiveness and bioavailability of bioactive compounds. Such a finding indicates a promisiçng role for nanoparticle technology in optimizing therapeutic strategies. The findings of our study align with previous literature, affirming the vital role of P2X7R in the modulation of the inflammatory response. For instance, studies by Cui et al. (Cui et al. 2020) and Pelegrin et al. (Pelegrin et al. 2008) demonstrated that RV has an inhibitory effect on P2X receptors, with a subsequent reduction in inflammation and tissue damage. This signals that RV and particularly AgNP-RV could serve as potential tools in mitigating tissue damage and inflammatory response in sepsis.

## Conclusion

In conclusion, this study highlights the promising therapeutic potential of silver nanoparticle–enhanced resveratrol in combatting sepsis-induced lung injury, marking a significant contribution to the current body of research. The significant improvements observed in key markers of inflammation and apoptosis, along with the modulation of oxidative status, shed light on the profound, protective effects of these compounds. It is plausible that the potent antioxidant characteristics of resveratrol and AgNP-RV might underpin these effects, yet their significant role in mitigating the harmful inflammation cascade, specifically by suppressing P2X7R and associated cytokine responses, cannot be understated. This offers an innovative perspective on preventing the detrimental pulmonary damage associated with sepsis. Hence, the introduction of resveratrol and AgNP-RV may pave the way for novel therapeutic interventions in the realm of sepsis management.

## Data Availability

No datasets were generated or analysed during the current study.
